# Whole plastome sequence of *Hibiscus moscheutos* L. 1753 (Malvaceae) and its phylogenetic analysis

**DOI:** 10.1080/23802359.2025.2466604

**Published:** 2025-02-18

**Authors:** Yu Sun, Yutian Jin, Maoyin Sun, Liqiang Wang, Qianqian Liu

**Affiliations:** a College of Pharmacy, Heze University, Heze, PR China; b School of Medicine, Nanjing University of Chinese Medicine, Nanjing, PR China

**Keywords:** *Hibiscus moscheutos*, Malvaceae, next-generation sequencing technology, phylogenetic analysis, whole plastome sequence

## Abstract

*Hibiscus moscheutos*, a perennial herb in the Malvaceae family, has medicinal properties but lacks molecular data. This study sequenced and analyzed its first complete plastome using next-generation sequencing. The 160,208 bp circular plastome has a typical quadripartite structure with 130 genes and 36.93% GC content. Phylogenetic analysis showed that *H. mutabilis* is sister to *H. taiwanensis*. This study provides essential molecular data for future research on *Hibiscus* phylogeny, taxonomy, and evolution.

## Introduction

*Hibiscus moscheutos* L. 1753 (Linnaeus 1753), commonly known as grass hibiscus or large-flowered okra, belongs to the Malvaceae family and is native to North America’s wetlands. The species thrives in warm, humid environments, preferring fertile sandy loam soils near water bodies, while also exhibiting some shade tolerance. Flowering occurs between June and August, and *H. moscheutos* is valued for its ornamental, edible, and medicinal properties (Chensom et al. 2020). Its flowers contain high concentrations of bioactive compounds such as flavonoids, anthocyanins, and phenolics, which display a range of biological activities, including antioxidant, cardioprotective, hypoglycemic, and antihypertensive effects. Anthocyanins are particularly noted for their potent antioxidant and anti-inflammatory properties, potentially mitigating risks associated with cardiovascular diseases, diabetes, arthritis, and cancer.

As a perennial species, *H. moscheutos* adapts to various environments and reproduces quickly (Snow et al. 2000), supporting ecological equilibrium. The plant also has the capacity to absorb harmful substances and purify the environment. Through its robust water and nutrient absorption capacity (Shimamura et al. 2007), *H. moscheutos* effectively reduces soil erosion and improves soil quality, making it a valuable species for ecological restoration and environmental protection.

Despite its ecological and medicinal importance, the complete plastome of *H. moscheutos* has not yet been published, although a preliminary version is available in GenBank with accession number ON007127.1. In this study, the complete plastome of *H. moscheutos* was assembled and characterized using Illumina paired-end sequencing data, providing a critical resource for future research in conservation and phylogenetics.

## Materials and methods

**Figure 1.**
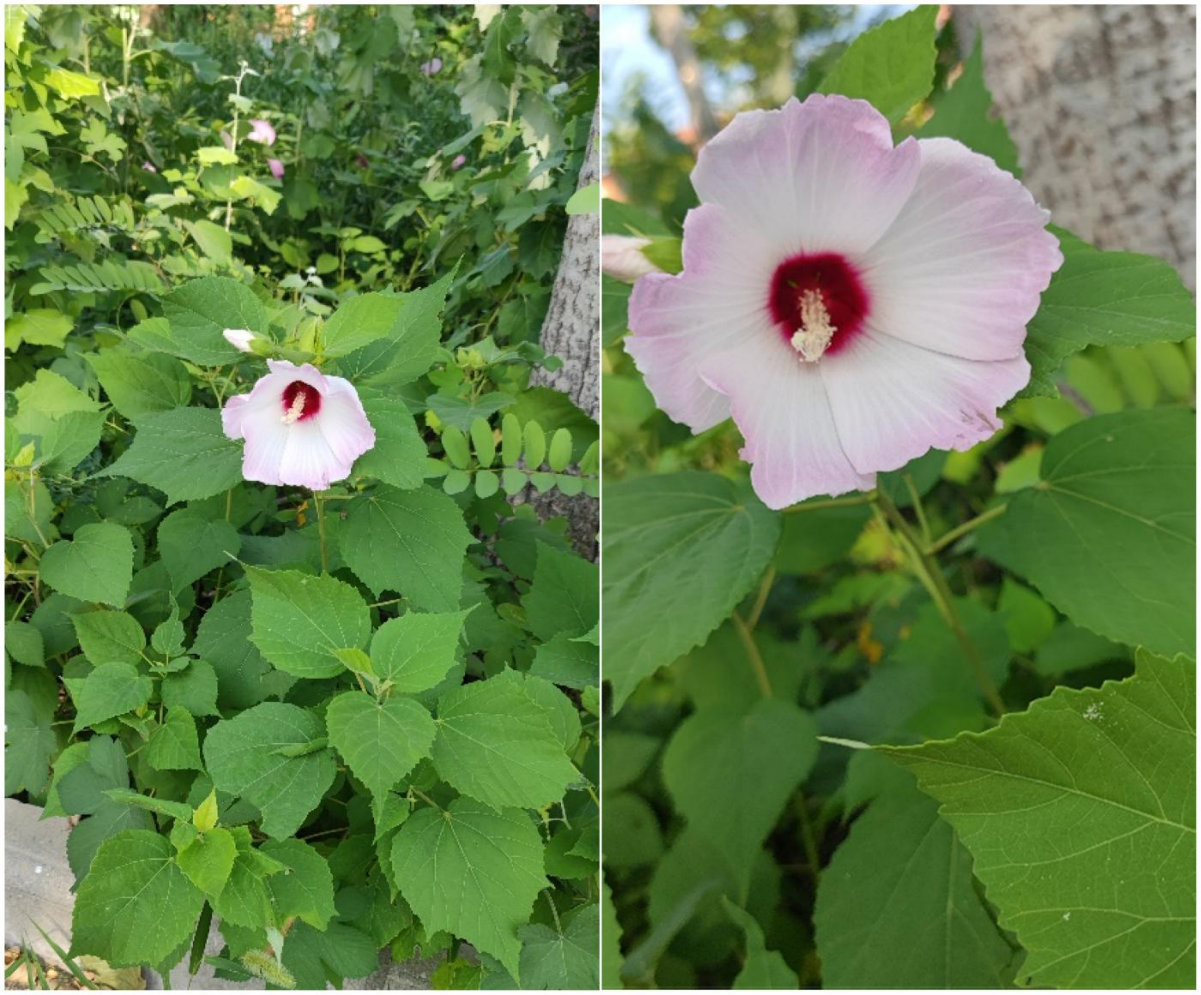
Plant morphology of *Hibiscus moscheutos*. Photographs were taken by Liqiang Wang at coordinates 35°16′11.78″N, 115°28′0.19″E. Key identifying characteristics of the species: perennial herbs, growing 1–2.5 m in height, with stellate pilose or nearly glabrous texture. Leaves are ovoid or ovate-lanceolate, 7–18 cm long, 4–8 cm wide, subglabrous or finely pilose on the upper surface, and covered with gray-white felt-like hairs on the lower surface, with obtuse crenate edges. Flowers are solitary in leaf axils at branch tips, with a pedicel length of 2–8 cm, small linear bracts, and a campanulate calyx with five ovate-triangular lobes. The corolla is white, pink, or red, with obovate flowers valves, and the inner face is basally bearded. Fruit is a conical-ovoid capsule, 2.5–3 cm long, subrounded reniform.

Samples of *H. moscheutos* (Figure 1) were collected from the campus of Heze University, located in Heze City, Shandong Province, China (35°16′11.78″N, 115°28′0.19″E). A voucher specimen was deposited in the Herbarium of Heze University under specimen number HZ220826 (contact: Liqiang Wang, lys832000@163.com).

Tender leaves from healthy plants were used for the extraction and purification of whole genomic DNA, utilizing the Plant Genomic DNA Kit (Tiangen Biotech, Beijing, China). The extracted DNA was assessed for quality and fragmented into ∼300 bp inserts. Library preparation and sequencing were carried out on the Illumina NovaSeq 6000 platform by Wuhan Benagen Technology Co. (Wuhan, China), resulting in approximately 11.1 GB of clean reads (FASTQ format) after removing adapters and low-quality bases.

Plastome assembly was performed using GetOrganelle (v1.7.1) (Jin et al. 2020), with annotation done through CPGAVAS2 (Shi et al. 2019) and subsequently refined manually using Apollo (Pontius 2018). The annotated plastome was then submitted to GenBank via BankIt. A circular genome map was generated using CPGview (Liu et al. 2023). For phylogenetic analysis of *H. moscheutos*, plastome sequences from 10 other *Hibiscus* species and one outgroup species (*Melia azedarach*) were aligned using MAFFT (Katoh and Standley 2016) with default parameters (https://mafft.cbrc.jp/alignment/software/). A maximum-likelihood (ML) phylogenetic tree was constructed with IQ-TREE (v2.0) (Nguyen et al. 2015), using 1000 bootstrap replicates and the K3Pu + F + I + R4 substitution model, selected based on the Bayesian information criterion (BIC) score.

## Results

**Figure 2.**
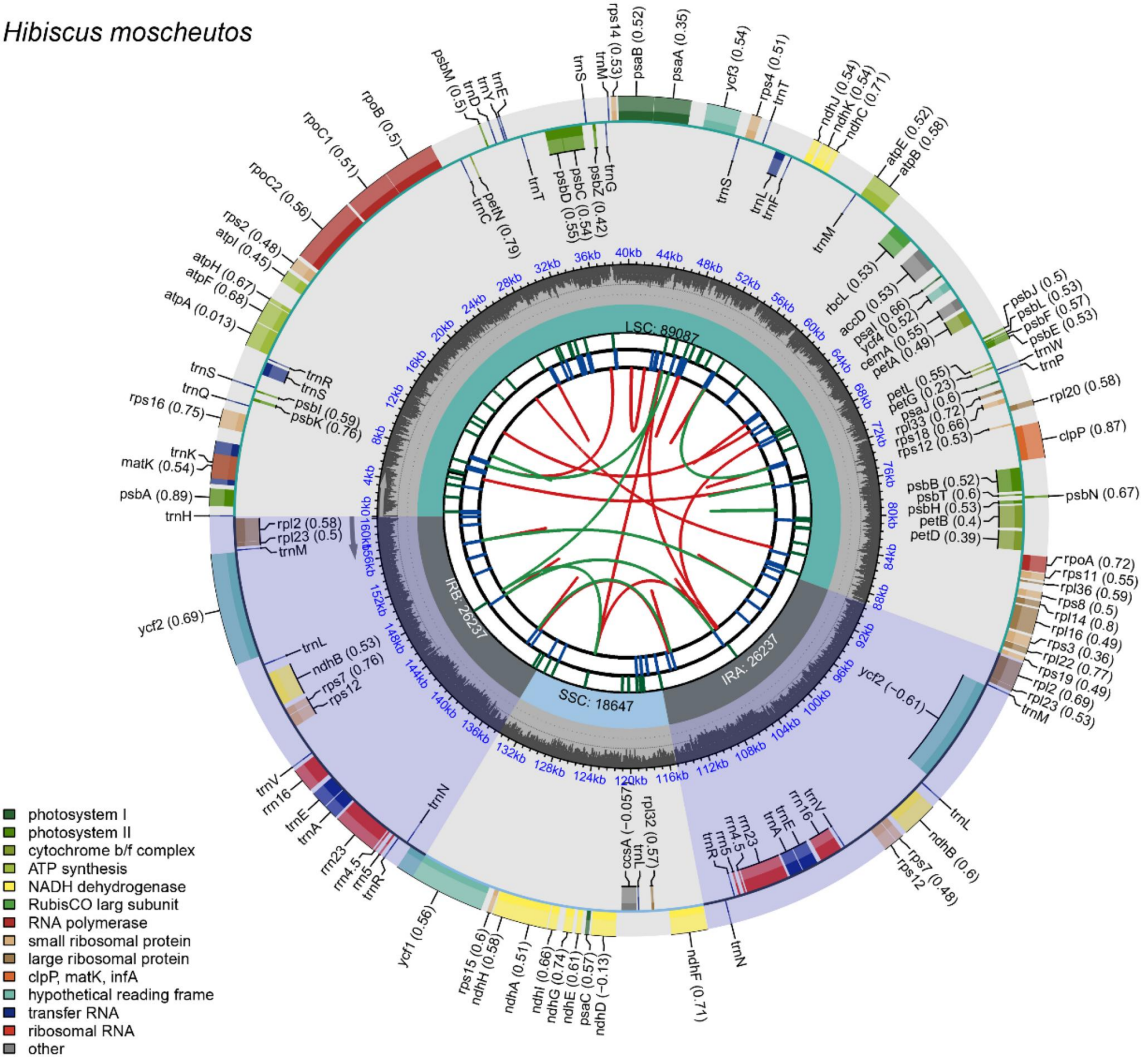
Plastome mapping of *Hibiscus moscheutos*. The species name appears in the top left corner. The map contains six tracks by default. From the center outward, the first track shows dispersed repeats, including direct and palindromic repeats connected by red and green arcs. The second track displays long tandem repeats as blue bars, while the third displays short tandem repeats or microsatellite sequences in varying colors. The fourth track displays the SSC, IRa, IRb, and LSC regions. The fifth track shows the GC content along the genome. The sixth track presents various genes, color-coded according to their functional classification (shown in the bottom left corner). For protein-coding genes, letters after gene names specify subunits or family members; numbers indicate gene variants or functional differentiation. For rRNA genes, numbers after gene names represent rRNA size in Svedberg units. For tRNA genes, letters after gene names indicate amino acids they recognize. Genes of unknown function are indicated with numbers corresponding to different hypothetical coding genes. Inner genes are transcribed clockwise, while outer genes are transcribed anticlockwise.

Sequencing and assembling the plastome of *Hibiscus moscheutos* produced a high-quality genome sequence, which was annotated and thoroughly analyzed. The plastome is 160,208 bp in length and features a typical tetrameric structure (Figure 2). It consists of a large single-copy (LSC, 89,087 bp) region, a small single-copy (SSC, 18,647 bp) region, and two inverted repeat (IRa and IRb, 26,237 bp each) regions. The average, maximum, and minimum coverage depths at each position in the assembled genome were 2030.20×, 3353×, and 244×, respectively (Figure S1).

The plastome encodes 130 genes, including 85 protein-coding genes, eight rRNA genes, and 37 tRNA genes, with a guanine-cytosine (GC) content of 36.93%. Of the annotated genes, 20 contain one or two introns and are classified as cis-splicing genes. Genes such as *rps*16, *atp*F, *rpo*C1, *pet*B, *pet*D, *rpl*16, *rpl*2 (×2), *ndh*B (×2), *ndh*A, *trn*K-UUU, *trn*S-CGA, *trn*L-UAA, *trn*E-UUC (×2), and *trn*A-UGC (×2) each contain one intron (Figure S2). The genes *clp*P and *ycf*3 each have two introns (Figure S2). The *rps*12 gene is a trans-splicing gene, with its downstream 3′ end located in the IR regions and containing an intron in each 3′ end (Figure S3).

**Figure 3.**
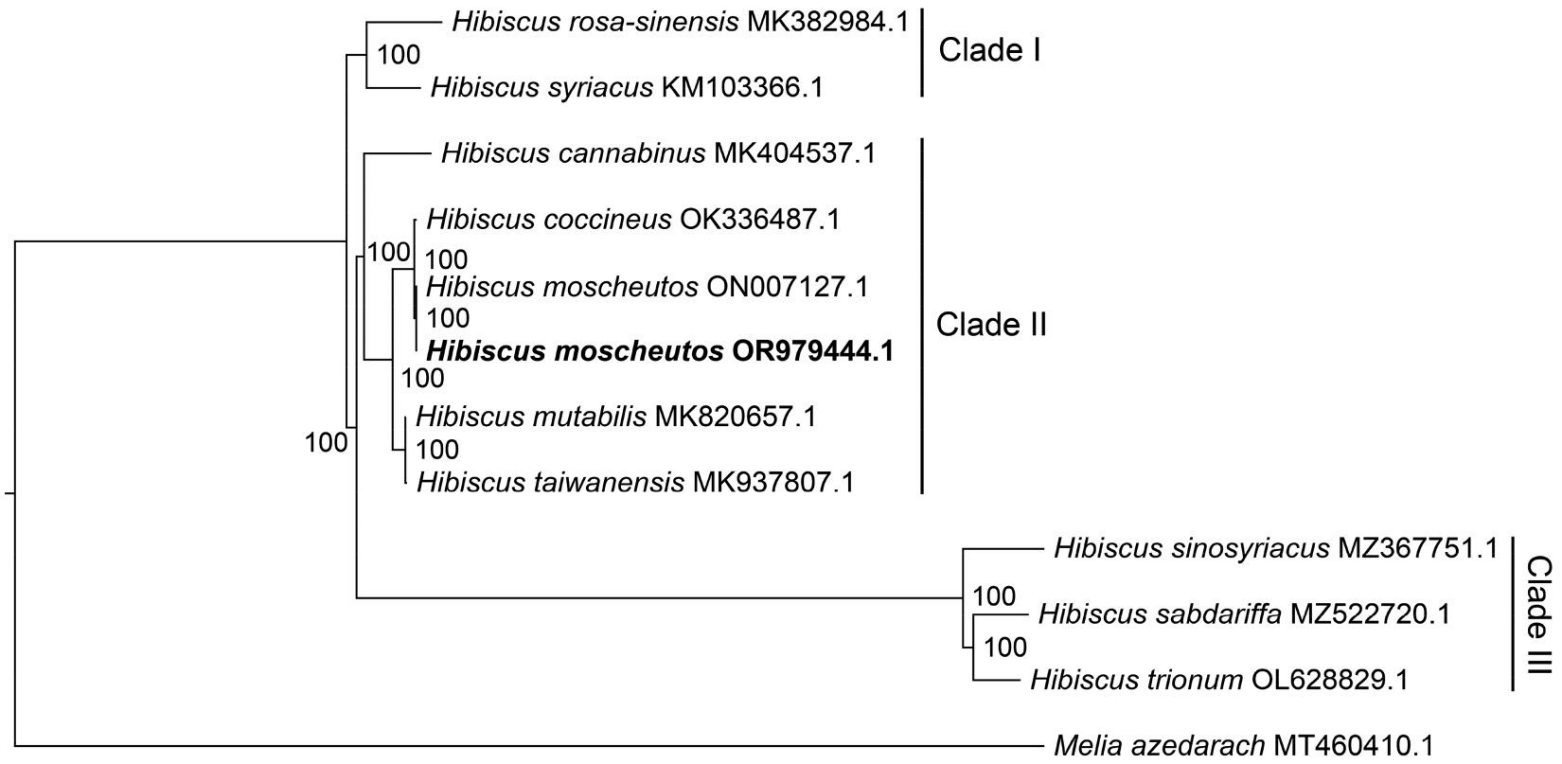
Maximum-likelihood (ML) phylogeny of *Hibiscus moscheutos* based on complete plastome sequences. Bootstrap values based on 1000 replicates are shown on each node. Eleven *Hibiscus* plant and one outgroup were used to reconstruct the ML tree, which are *H. rosa*-*sinensis* (MK382984.1, Abdullah et al. 2020), *H. syriacus* (KM103366.1), *H. cannabinus* (MK404537.1), *H. coccineus* (OK336487.1, Wang et al. 2022), *H. moscheutos* (ON007127.1), *H. moscheutos* (OR979444, this study), *H. mutabilis* (MK82065.1, Abdullah et al. 2020), *H. taiwanensis* (MK937807.1, Go et al. 2024), *H. sinosyriacus* (MZ367751.1, Kwon et al. 2023), *H. sabdariffa* (MZ522720.1, Kwon, Park, et al. 2022), *H. trionum* (OL628829.1, Kwon et al. 2022), and *Melia azedarach* (MT460410.1, outgroup). The new *H. moscheutos* plastome in this study is highlighted in bold font.

To examine the phylogenetic relationships of *H. moscheutos*, complete plastome sequences from 11 *Hibiscus* species and an outgroup were used to construct a ML tree. Most nodes in the tree received strong support with 100% bootstrap values (Figure 3). In the tree, two *H. moscheutos* plants (OR979444.1 and ON007127.1) formed a monophyletic branch and were most closely related to *H. coccineus* (Figure 3). The *Hibiscus* species in the tree were classified into three clades: Clade I included *H. rosa-sinensis* and *H. syriacus*; clade II consisted of *H. cannabinus*, *H. coccineus*, *H. moscheutos*, *H. mutabilis*, and *H. taiwanensis*; and clade III comprised *H. sinosyriacus*, *H. sabdariffa*, and *H. trionum* (Figure 3).

## Discussion and conclusions

In this study, the plastome of *H. moscheutos* was assembled using short-read data. The genome size, GC content, and gene composition of the plastome are similar to those of other *Hibiscus* species (Abdullah et al. 2020; Kwon, et al. 2022; Kwon et al. 2022; Wang et al. 2022; Kwon et al. 2023; Go et al. 2024). However, differences in sequence length, gene number, and structural features were observed between species. These variations could reflect specific adaptations to diverse environmental factors, such as cold and heat tolerance (Barrios and Ruter 2019). In short, this study presents valuable information for further exploration of the biological characteristics, genetic diversity, and evolutionary development of *H. moscheutos*.

The phylogenetic analysis indicated that two *H. moscheutos* plants and *H. coccineus* formed a monophyletic clade, which then branched with other closely related species, such as *H. mutabilis* and *H. taiwanensis*. Some of the topological results were consistent with previous studies. For example, clade I, which includes *H. rosa-sinensis* and *H. syriacus*, was consistent with earlier findings (Xu et al. 2019; Hu et al. 2021; Wang et al. 2022). Additionally, *H. mutabilis* and *H. taiwanensis* clustered together, as reported by Li et al. (2022). The phylogenetic analysis was strongly supported by bootstrap values, providing robust evidence for understanding the evolutionary relationships and history of *H. moscheutos*. These findings offer a scientific foundation for the conservation and utilization of this plant resource.

## Supplementary Material

Supplemental Material

## Data Availability

The complete chloroplast genome sequence of *Hibiscus moscheutos* in this study has been submitted to the NCBI database under the accession number OR979444.1 (https://www.ncbi.nlm.nih.gov). The associated BioProject, BioSample, and SRA numbers are PRJNA1166703, SAMN41918793, and SRR29471945, respectively.
