# Supplementary material for: Whole plastome sequence of Hibiscus moscheutos L. 1753 (Malvaceae) and its phylogenetic analysis
Source: Mitochondrial DNA B Resour. 2025 Feb 18;10(3):239–43. doi: 10.1080/23802359.2025.2466604 (PMC11841096; doi:10.1080/23802359.2025.2466604)
Supplement: Supplemental Material [file TMDN_A_2466604_SM3640.docx]

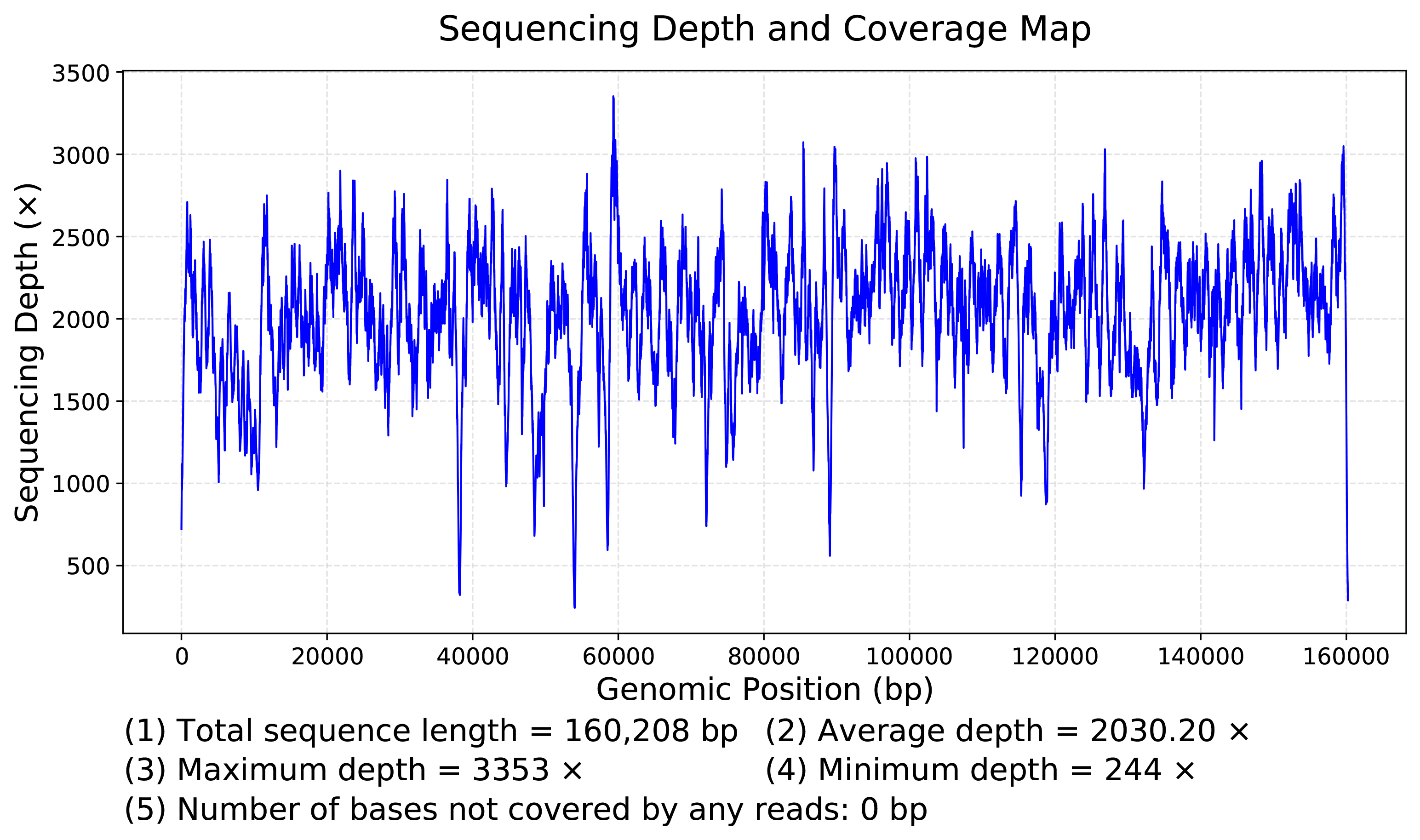


**Figure S1.** Depth of coverage map of the plastome of *Hibiscus sabdariffa*. The horizontal coordinate is the positional information of the nucleotide. The vertical coordinate is the coverage depth at each position of the plastome, which was calculated by minimap2 (Li 2018) and samtools (Li *et al*. 2009).


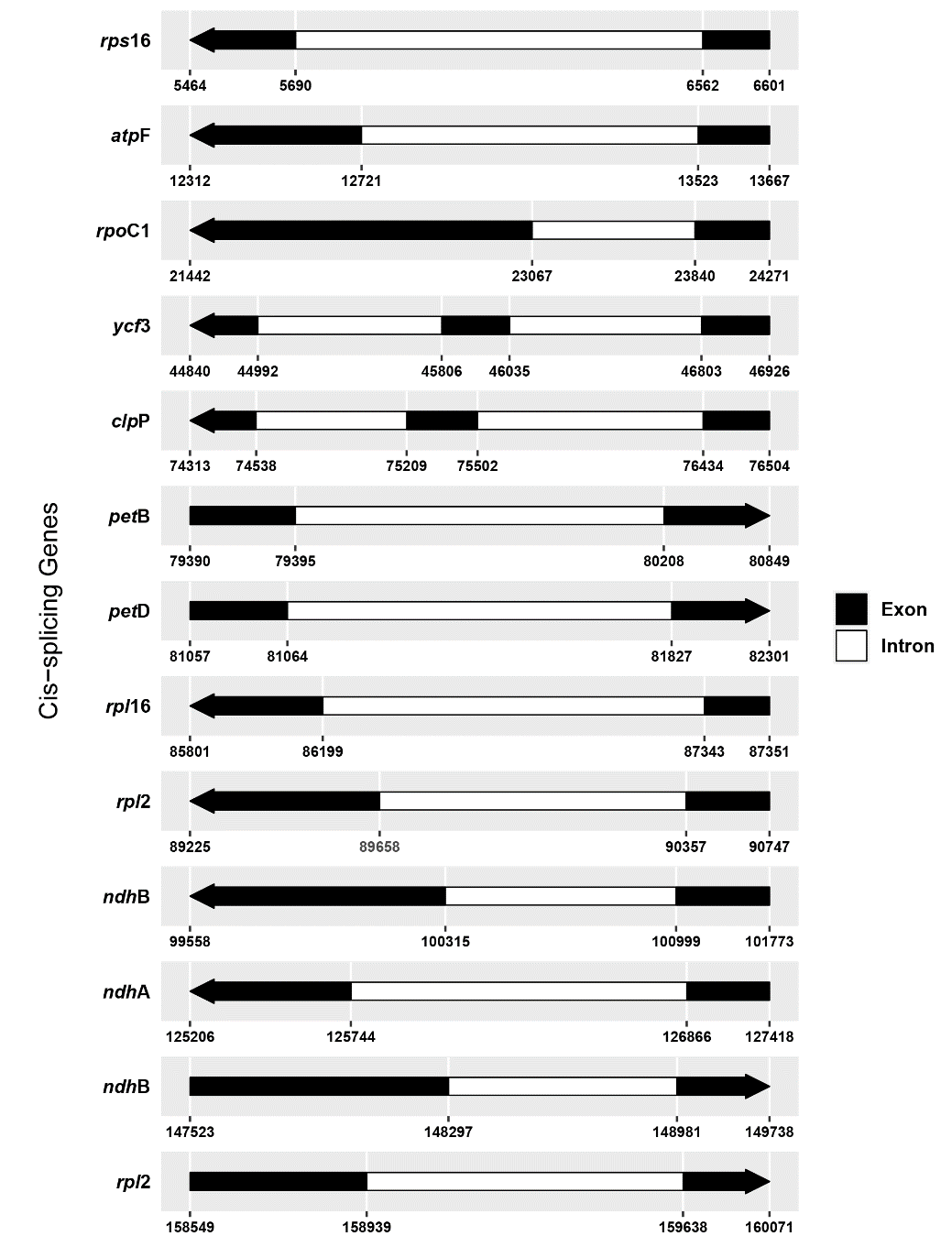


**Figure S2.** Diagram of cis-splicing genes within the plastome of *Hibiscus moscheutos*. This diagram was created using CPGview (Liu *et al*. 2023) software.


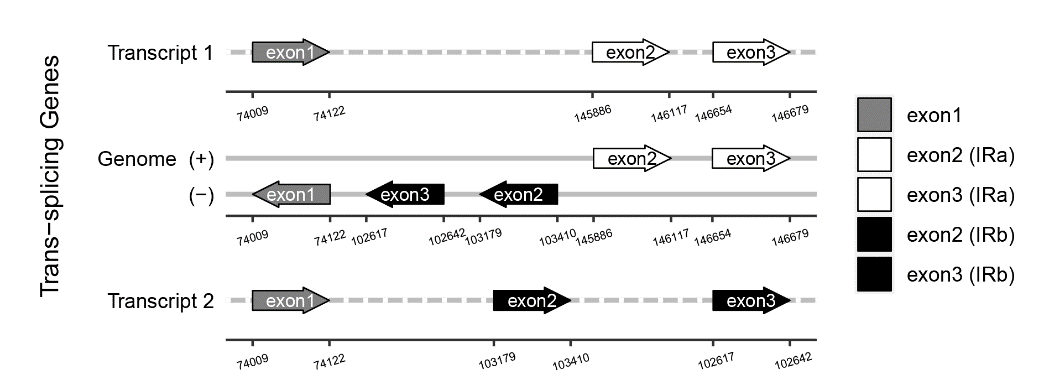


**Figure S3.** Diagram of the trans-splicing genes (*rps*12) in the plastome of *Hibiscus moscheutos*. The diagram was created using CPGview (Liu *et al*. 2023) software.
